# Apparent diffusion coefficient as it relates to histopathology findings in post-chemotherapy nephroblastoma: a feasibility study

**DOI:** 10.1007/s00247-017-3931-9

**Published:** 2017-07-01

**Authors:** Annemieke S. Littooij, Peter G. Nikkels, Christina A. Hulsbergen-van de Kaa, Cees P. van de Ven, Marry M. van den Heuvel-Eibrink, Øystein E. Olsen

**Affiliations:** 1 Department of Radiology and Nuclear Medicine, University Medical Centre Utrecht/Wilhelmina Children’s Hospital, Heidelberglaan 100, 3584 CX Utrecht, The Netherlands; 2 Department of Pathology, University Medical Centre Utrecht, Utrecht, The Netherlands; 3 Department of Pathology, Radboud University Medical Centre, Nijmegen, The Netherlands; 4 Department of Paediatric Surgery, Princess Maxima Centre for Paediatric Oncology, Utrecht, The Netherlands; 5 Department of Paediatric Oncology, Princess Maxima Centre for Paediatric Oncology, Utrecht, The Netherlands; 6 Department of Radiology, Great Ormond Street Hospital for Children, London, UK

**Keywords:** Apparent diffusion coefficient, Children, Diffusion-weighted imaging, Magnetic resonance imaging, Nephroblastoma, Wilms tumour

## Abstract

**Background:**

Nephroblastomas represent a group of heterogeneous tumours with variable proportions of distinct histopathological components.

**Objective:**

The purpose of this study was to investigate whether direct comparison of apparent diffusion coefficient **(**ADC) measurements with post-resection histopathology subtypes is feasible and whether ADC metrics are related to histopathological components.

**Materials and methods:**

Twenty-three children were eligible for inclusion in this retrospective study. All children had MRI including diffusion-weighted imaging (DWI) after preoperative chemotherapy, just before tumour resection. A pathologist and radiologist identified corresponding slices at MRI and postoperative specimens using tumour morphology, the upper/lower calyx and hilar vessels as reference points. An experienced reader performed ADC measurements, excluding non-enhancing areas. A pathologist reviewed the corresponding postoperative slides according to the international standard guidelines. We tested potential associations with the Spearman rank test.

**Results:**

Side-by-side comparison of MRI–DWI with corresponding histopathology slides was feasible in 15 transverse slices in 9 lesions in 8 patients. Most exclusions were related to extensive areas of necrosis/haemorrhage. In one lesion correlation was not possible because of the different orientation of sectioning of the specimen and MRI slices. The 25% ADC showed a strong relationship with percentage of blastema (Spearman rho=−0.71, *P*=0.003), whereas median ADC was strongly related to the percentage stroma (Spearman rho=0.74, *P*=0.002) at histopathology.

**Conclusion:**

Side-by-side comparison of MRI–DWI and histopathology is feasible in the majority of patients who do not have massive necrosis and hemorrhage. Blastemal and stromal components have a strong linear relationship with ADC markers.

## Introduction

Nephroblastoma (Wilms tumour) is the most common childhood renal malignancy. MR imaging is increasingly used for local staging [1]. Nephroblastomas develop from embryonic kidney cells, containing varying amounts of tissue that represent different stages of renal development (blastema, stromal and epithelial components). Classic triphasic nephroblastoma consists of variable amounts of each of these three cell lines [2]. There are two approaches in the treatment of nephroblastoma: The Children’s Oncology Group (COG) in North America advocates upfront surgery followed by chemotherapy, depending upon the histopathological result, whereas the International Society of Pediatric Oncology (Societe Internationale d’Oncology Pediatrique, or SIOP) in Europe focuses on using preoperative chemotherapy. Both approaches show equally high rates of overall survival [2]. However histopathology findings have different implications depending on whether the tumour is resected before or after chemotherapy. In the SIOP trial, nephroblastomas with diffuse anaplasia or blastema-type tumour are classified as high-risk, whereas in the COG system, the blastema component has less prognostic significance. The COG classification separates tumour into three categories: favourable histology (no anaplasia), focal anaplasia or diffuse anaplasia [2].

To improve care for these children, prognostic biomarkers for better risk stratification are needed to maximize survival with minimal toxicity. The ability to identify high-risk histopathological subtypes (such as blastema-predominant lesions after preoperative treatment) might enable more personalized treatment decisions in the future [2, 3]. The addition of diffusion-weighted imaging (DWI) to the standard MRI protocol might provide information regarding subtype characterisation and treatment response beyond necrosis and volume change [1, 4, 5].

Previous studies compared whole-tumour apparent diffusion coefficient (ADC) measurements with post-resection whole-tumour histopathological assessment [4, 5]. These studies reported a relationship between ADC markers and stromal subtype histopathology. Unfortunately both studies found relatively low ADC values in both epithelial and blastemal subtypes. However using a single ADC variable to represent the whole tumour could plausibly obscure underlying correlations between ADC value and histopathology type. Direct correlation of single-slice ADC measurements with a corresponding histopathology slice might therefore refine the analysis and enhance the predictive value of DWI–MRI.

The purpose of this study was to show the feasibility of correlating MRI–DWI results with post-resection histopathology by comparing ADC metrics to histopathological subtypes in nephroblastomas.

## Materials and methods

### Patients

All patients included in the current study gave informed consent for pseudonymized registration and analyses of data obtained by conducting the international diagnostic standard of care according to the International Society of Pediatric Oncology (SIOP) protocol. The Princess Maxima Centre for Paediatric Oncology has been the national referral centre for nephroblastoma in the Netherlands since November 2014. Between May 2015 and April 2016, 23 consecutive children at that institution were eligible for inclusion. Inclusion criteria were histologically proven nephroblastoma, treatment according to the SIOP protocol (preoperative chemotherapy followed by surgery) and availability of a complete MRI study including DWI after preoperative treatment, just before tumour nephrectomy. Exclusion criteria were severe artefacts at DWI or contrast-enhanced images, slice area less than 3 cm^3^ and predominant necrotic, haemorrhagic or cystic lesions. Although the presence of predominant cystic, haemorrhagic or necrotic components yield useful information, the ADC values of these components are clinically irrelevant. Moreover, the ADC measurements of these predominant cystic, haemorrhagic or necrotic lesions show a broader interobserver variablity [6].

### Magnetic resonance imaging

Contrast-enhanced MRI of the abdomen including DWI was performed on a 1.5-T system (Achieva; Philips Medical Systems, Best, the Netherlands). Coronal 3-D T2-W imaging along with fat-suppressed T1-W imaging before and after gadolinium-contrast-medium administration was acquired. Diffusion-weighted imaging was performed in axial plane during free-breathing, applying b values of at least 0 s/mm^2^, 100 s/mm^2^ and 1,000 s/mm^2^. The imaging parameters are displayed in Table 1. The ADC maps were calculated using a mono-exponential fit with b values 0 s/mm^2^ and 1,000 s/mm^2^. Depending on their ability to cooperate, children were awake (*n*=1) or under general anaesthesia (*n*=7). No oral contrast agents were used. Gadobutrol (Gadovist; Bayer Pharma, Berlin, Germany) was administered intravenously at a dose of 0.1 mmol/kg body weight. Hyoscine butylbromide (Buscopan; Boehringer Ingelheim, Bracknell, UK) was administered at an intravenous dose of 0.4 mg/kg body weight (with a maximum of 5 mg in children younger than 6 years) to reduce the peristaltic artefacts. All children were screened for MR contraindications, intravenous contrast agent contraindications, and intravenous hyoscine butylbromide contraindications.

**Table 1:** Scan parameters at 1.5-T MRI for imaging nephroblastoma after neoadjuvant chemotherapy

Parameter	T2-W SPIR	3-D T2-W	DWI	T1-W pre/post
Pulse sequence	2-D spectral presaturation with inversion recovery spin echo	3-D turbo spin-echo with variable flip angle	2-D single-shot spin echo with spectral fat saturation	2-D ultrafast spoiled gradient echo with fat-suppression
Repetition time (ms)	1,820	447	1,946	5.50
Echo time (ms)	80	90	76	2.70
Slice orientation	Axial	Coronal	Axial	Axial
Slice thickness (mm)	5	1.15	5	3
Slice gap (mm)	1	0	0	1
Echotrain length	30	85	35	60
Acquisition matrix	288 × 60	348 × 348	88 × 70	232 × 233
Receiver bandwidth (Hz/pixel)			2,534	
b values (s/mm^2^)	-	-	At least 0, 100, 1000	-

*DWI* diffusion-weighted imaging*, SPIR* spectral presaturation inversion recovery

### Direct correlation

**Fig. 1:**
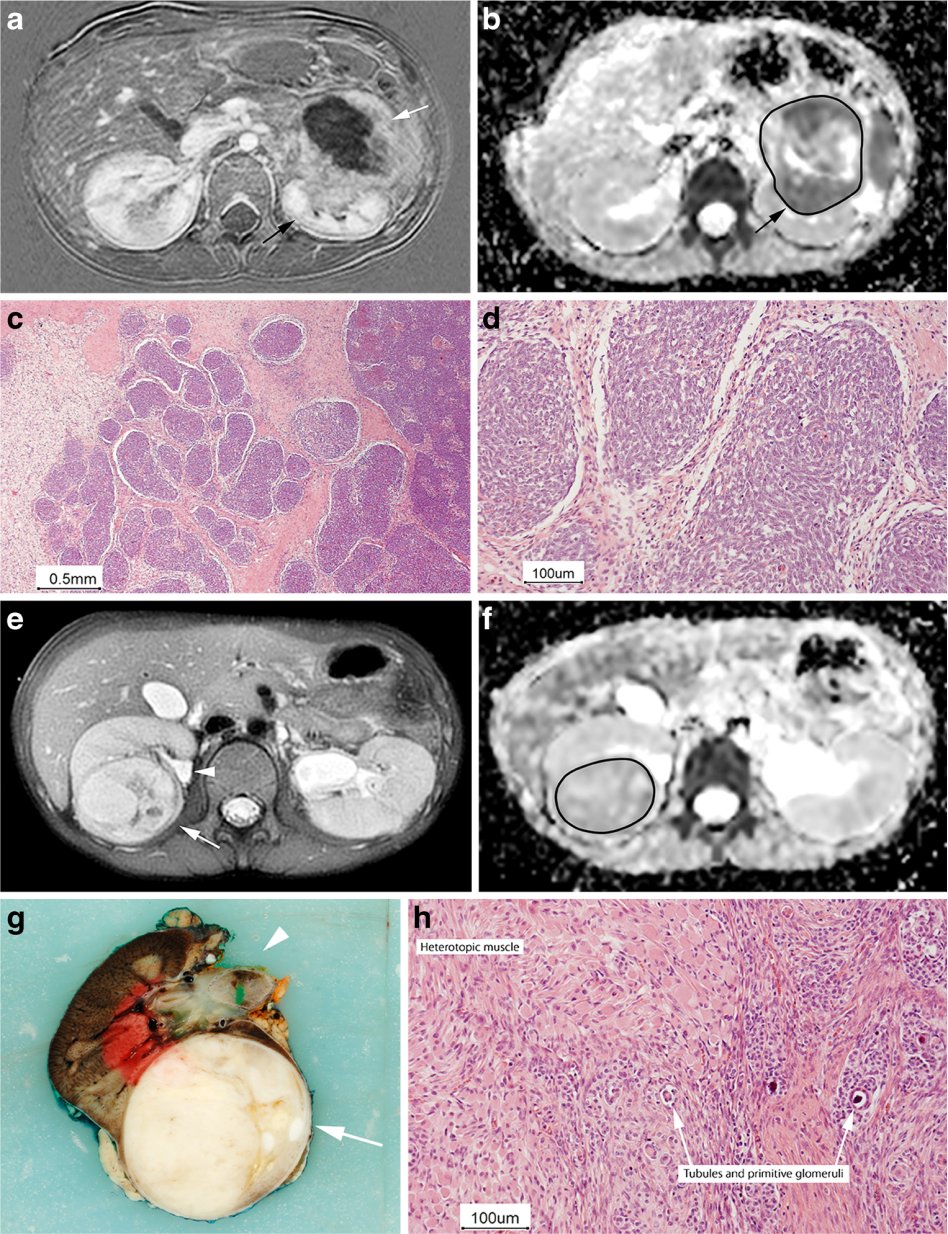
Bilateral nephroblastoma in a 4-year-old boy. **a, b** MR imaging, left lesion. Parallel axial subtracted post-contrast T1-W MR image (**a**) and apparent diffusion coefficient (ADC) map (**b**) after preoperative treatment show an infiltrative growing tumour central in the left kidney. Besides a central non-enhancing part (**a**, *white arrow*), the remaining viable tumour (*black arrow*) shows a relatively low ADC value (median ADC 0.99 × 10^−**3**^ mm^**2**^/s). After left partial nefrectomy, the histopathological review showed 60% chemotherapy-induced changes and 75% blastema in the viable part of the tumour. **c, d** Histology, left lesion. The hematoxylin and eosin stain with low-power magnification (**c**) and high-power magnification (**d**) shows an abundant blastemal component that contains small cells with hyperchromatic nuclei and scant cytoplasm**.** Right nephrectomy was performed a few weeks later. **e, f** MR imaging, right lesion. In contrast to the left side, the right lesion is rather homogeneous [(**e)**; axial T2-W image with fat suppression], with relative high ADC values [(**f)**; ADC 1.20 × 10^−**3**^ mm^**2**^/s]. **g, h** Pathology, right lesion. Corresponding histopathological review showed 10% chemotherapy-induced changes and 94% stromal component in the viable part of tumour. During side-by-side matching we used the tumour morphology (*arrow*) and anatomical landmarks (e.g., pyelum; *arrowhead* in (**g**). The hematoxylin and eosin stain with high-power magnification shows heterotopic muscle cells with scattered tubules and primitive glomeruli (**h**, *arrows*)

The dorsal and ventral side and hilar region of the post-resection specimens were marked with different-colour dyes according to instructions by the involved surgeon. The specimens were sectioned in successive 10- to 15-mm transverse slices in a cranial to caudal sequence. One specimen was fixated in agar and lamellated in 4-mm slices (Fig. 1). The photographs of the pathological slices were matched with the corresponding T2-W or post-contrast T1-W images at MRI by a radiologist (A.S.L., 10 years of experience with paediatric body MRI) in conjunction with the pathologist (P.G.N., 20 years of experience with paediatric histopathology). The closest corresponding transverse image and pathological macroslice were paired on the basis of anatomical and tumour landmarks, using the upper and lower calyces and hilar vessels as reference points.

### Apparent diffusion coefficient measurements

Anonymised MR datasets were transferred to DICOM software Osirix version 5.5.2 (Pixmeo SARL; Bernex, Switzerland). The paediatric radiologist (A.S.L.) performed ADC measurements at the predefined slices. The reader was masked to clinical patient data and histopathology reports. Freehand regions of interest were carefully drawn on the map using the available T1- and T2-W images to guide the outline of the entire tumour. We excluded areas with very low or no enhancement because these areas are thought to represent necrosis, haemorrhage or cystic elements that can skew the ADC parameters if included in the analysis. The intra- and interobserver variability of this method has been studied [6]. Therefore it was considered sufficient to have one reader.

### Histopathological review

At our institution all renal resection specimens are reviewed by the local histopathologist. Independent national SIOP pathology review (C.A.H.-vdK., 25 years of experience in paediatric oncology and uropathology) is performed for every paediatric renal tumour within 1 week according to the SIOP standard. The local paediatric histopathologist (P.G.N., 25 years of experience in paediatric and perinatal pathology) reviewed the predefined histological sections. The percentage of chemotherapy-induced changes and percentage of stromal, epithelial and blastemal elements was recorded, according to the SIOP definitions [7]. The subtype classification by the local pathologist was concordant with the central review in the included cases.

### Statistical analysis

Median and 25th percentile ADC were calculated for each slice. The association between the ADC values and percentage of subtypes at histopathology was assessed with Spearman rank correlation coefficient analysis, with r categorized correlations of 0–0.19=very weak, 0.2–0.39=weak, 0.40–0.59=moderate, 0.6–0.79=strong and 0.8–1.0=very strong. *P*-values less than 0.05 were considered statistically significant. Statistical analyses were performed with Statistical Package for the Social Sciences for Mac (SPSS, IBM, Armonk, NY).

## Results

### Patients

A total of 23 children were eligible for inclusion. Fifteen were excluded for the following reasons: no preoperative MRI avialable (*n*=3), extensive tumour haemorrhage or necrosis (*n*=8), incomplete MRI (*n*=3), direct correlation impossible (*n*=1). The final series consisted of the remaining 8 patients (mean age 4.4 years, range 2.2 years to 7.2 years, 1 male, Table 2). Nine tumours in these eight patients were included for analysis. Fifteen corresponding slices could be identified at MRI–DWI and histopathology.

**Table 2:** Characteristics of included patients (*n*=8)

Characteristics		
Gender *(n)*		
Male	1	
Female	7	
Age *(y)*		
Mean ± SD	4.4 ± 1.6	
Range	2.2–7.2	
Pathological subtype *(n=9)*		
Mixed	4	
Stromal	2	
Regressive	1	
Blastema	2	
Disease stage		Preoperative chemotherapy
II	2	AV × 4 wks
III	2	AV × 4 wks
IV	3	AVD × 6 wks
V	1	LT: AV × 8 wks RT: AV × 8 wks + AVD + CE

*AV* dactinomycin/vincristine, *AVD* dactinomycin/vincristine/doxorubicin, *CE* carboplatin/etoposide, *LT* left renal lesion, *RT* right renal lesion, *SD* standard deviation, *wks* weeks

### Feasibility of side-by-side comparison

One of 23 eligible patients was excluded related to different orientation of the slicing between the specimen and the MR images. This was probably related to a large posterior tumour arising from the lower pole of the left kidney that pushed the remaining kidney in a more horizontal position at MRI.

Figure 1 illustrates the matching of MR and nephrectomy specimen on the basis of the combination of anatomical landmarks (renal pelvis) and tumour morphology. The right nephrectomy specimen was fixated in agar and lamellated in 4-mm thick slices.

**Fig. 2:**
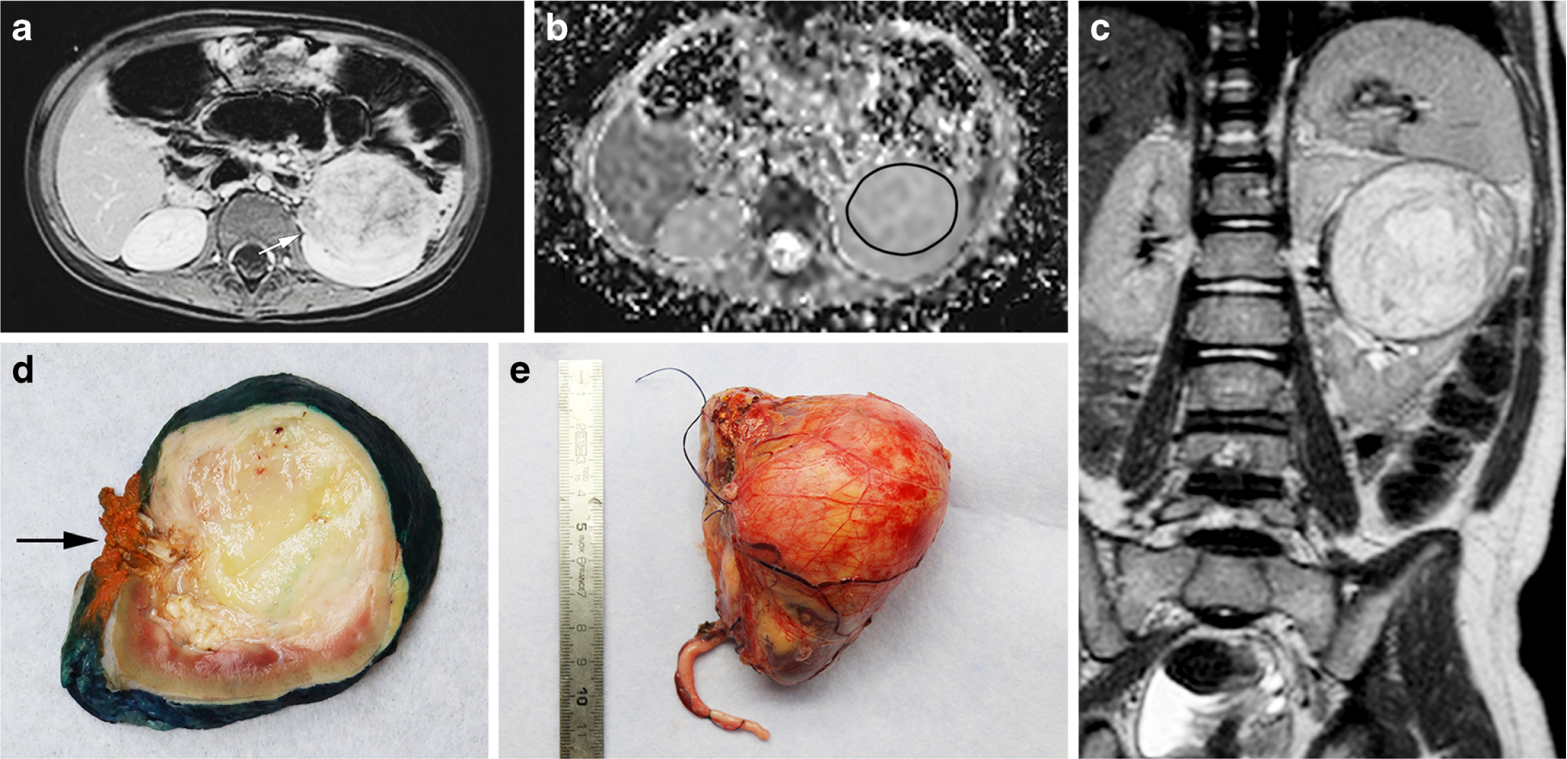
Large left-side renal tumour, found to be a histopathologically proven nephroblastoma, mixed subtype, in a 3-year-old girl. **a, b** MR imaging after pre-surgical treatment. Axial post-contrast T1-W MR image (**a**) and corresponding axial apparent diffusion coefficient (ADC) map (**b**) show a homogeneous tumour of the left kidney with median ADC value of 1.23 × 10^−**3**^ mm^**2**^/s. Tumour extends into a renal venous vessel (**a**, *arrow*). **c–e** Imaging and histology after pre-surgical treatment. Coronal 3-D T2-W image after preoperative treatment (**c**) together with the macroslide (**d**, *arrow*) points to the orange inked renal hilus) and gross histopathological specimen (**e**) illustrate the tumour in the mid-pole of the left kidney. Histopathological analysis of this slice showed 4% chemotherapy-induced changes and 84% stromal subtype histopathology in the viable part of this tumour slice. *ADC* apparent diffusion coefficient

Figure 2 shows the step section post-nephrectomy specimen for which the MR images were matched to the corresponding slice on the basis of the location of the renal hilum (including tumour thrombus in a side branch of the renal vein).

### Apparent diffusion coefficient parameters and subtype proportions

**Fig. 3:**
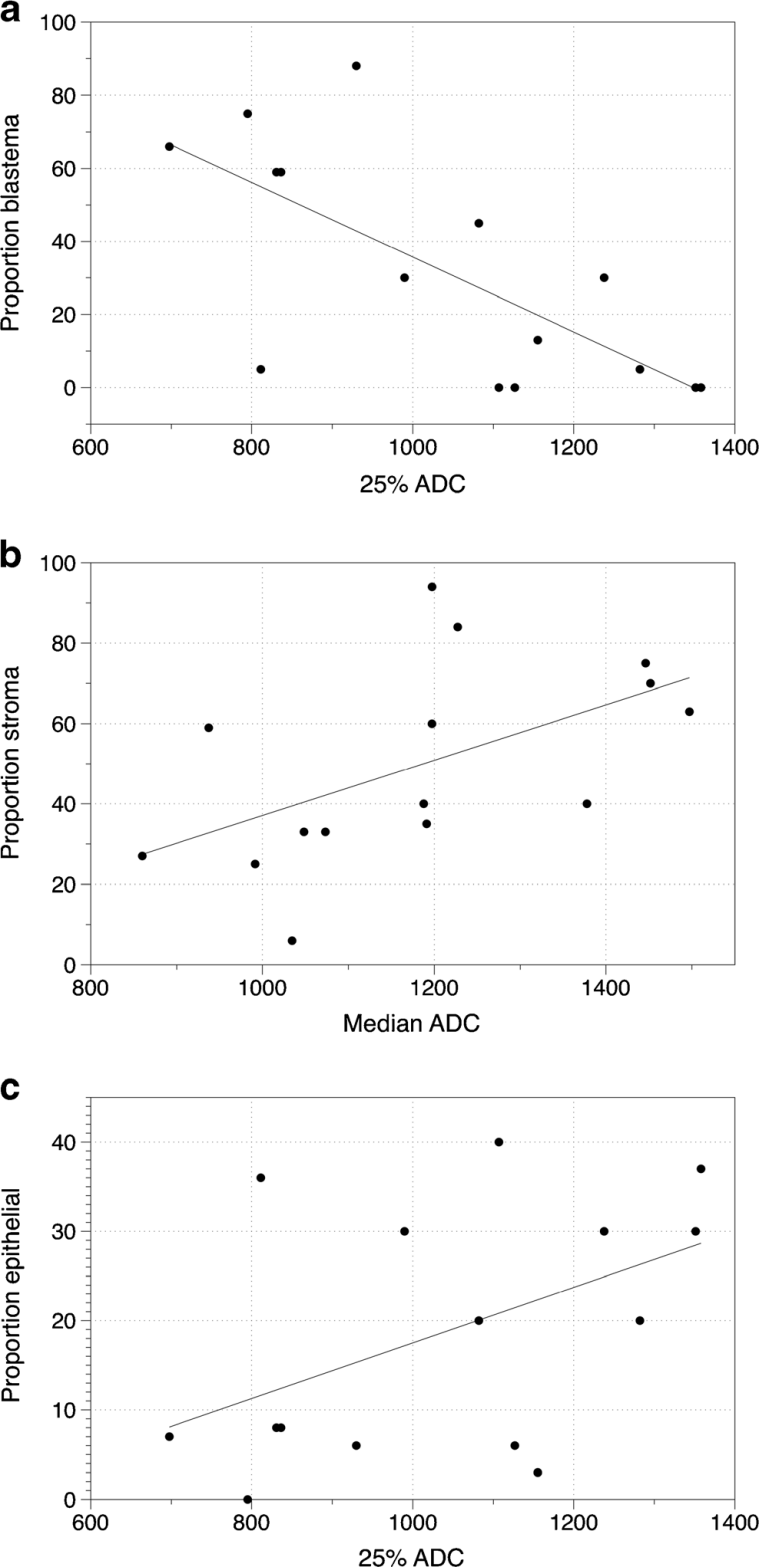
Scatter plots with correlation lines show the relationships between blastemal proportion and 25th percentiles apparent diffusion coefficient (ADC) (× 10^−**3**^ mm^**2**^/s) (**a**), stromal proportion and median ADC (× 10^−**3**^ mm^**2**^/s) (**b**) and epithelial proportion and median ADC (× 10^−**3**^ mm^**2**^/s) (**c**). A strong linear inverse relationship between proportion of blastemal components and 25th percentile ADC after preoperative treatment is nicely illustrated with the scatterplot (**a**). The strong linear relationship between proportion of stromal components and median ADC is illustrated in (**b**). There is no significant correlation between proportion epithelial components and median ADC value (**c**)

Scatter plots show the distribution of ADC values and the percentages of histopathological subtypes (Fig. 3). The 25th percentile ADC and the percentage of blastema in the 15 slices showed a strong inverse linear relationship (*r*=−0.71, *P*=0.003). Likewise, the median ADC and percentage of stroma were strongly linearly related (*r*=0.74, *P*=0.002). With the small number of lesions we included, there were no lesions with more than 40% epithelial components at histopathology. The percentage of epithelial component and median ADC showed no significant relationship (*r*=0.370, *P*=0.174).

## Discussion

Our explorative pilot study shows that direct correlation between MR images and post-resection histopathology specimens is feasible in the majority of lesions. However half of the potential patients were excluded because of extensive cystic, haemorrhagic or necrotic changes that occurred during preoperative treatment. With this limited number of lesions, we found a strong linear relationship between the stromal proportion at histopathology and median ADC. Our results replicate previous studies in 25 patients [4, 5]. However with side-by-side comparison of MR images and histopathology findings, the current reported linear relationship is more apparent. Likewise, there was a strong inverse linear relationship between percentage of blastema at histopathology and the lower quartile ADC. Our previous reported study found a similar, however weak, relation. Unfortunately only limited proportions of the epithelial subtype were present in our current reported study. Therefore potential linear relationship between 25th percentile ADC and percentage of blastemal components might be overestimated because both blastema and epithelial predominant lesions demonstrate relatively low ADC values [4, 5]. Furthermore the overall effect in a group of tumours cannot be directly extrapolated to the individual patient. If there is a significant overlap in ADC values, a single ADC value might be of limited clinical value for the individual patient. Therefore, reliable differentiation between epithelial- and blastemal-predominant lesions at presentation is probably not possible with ADC measurements alone. However identification of a considerable proportion of relatively low ADC values at presentation could guide tumour biopsy. With the combination of MRI–DWI findings and tumour biopsy results at presentation, the high-risk predominant blastemal subtype nephroblastoma could potentially be identified to help guide personalized treatment decisions [2].

The strong linear relationship between stromal histopathology and median ADC could be of additional clinical value, particularly in bilateral disease. In bilateral disease, accurate assessment of treatment response is important to direct therapy planning in order to spare as much renal function as possible. Second-line treatment chemotherapy is added or substituted when the tumour shrinkage appears poor [8]. However, following chemotherapy stromal-predominant nephroblastomas tend to differentiate into more mature stromal or mesenchymal tumor types [8]. Median ADC measurements could be a promising tool to identify stromal-predominant tumours that could respond to preoperative treatment with differentiation instead of shrinkage.

Previous studies have suggested that blastema is the most responsive tumour component to chemotherapy [9, 10]. Moreover, chemo-resistant blastemal subtype after preoperative treatment has prognostic value with respect to event-free and overall survivial [1, 2]. MRI with DWI might be of value to assess both tumour response to treatment and ADC values in residual viable parts of lesions [5]. The combination of evident tumour shrinkage or necrosis with relative low ADC values in viable tumour components could be suggestive of chemo-resistant blastema [4, 5]. Therefore identification of residual blastemal components with DWI might guide treatment decisions, including surgical planning of a nephron-sparing approach in bilateral disease.

Further prospective studies with a larger cohort of children should be performed to validate our preliminary findings. To achieve reliable side-by-side comparison between MRI findings and histopathology, a collaborative effort is essential. First, effective information transmission from surgeon to histopathologist during handling of the post-resection specimen is required. Furthermore, review of sliced specimen needs input from radiologist and histopathologist. In this explorative study, we have shown that with these efforts, side-by-side comparison is feasible.

Our study has several limitations. First, because of its retrospective nature, a selection bias could have been introduced, which weakens the general validity of our data. Second, an interpretation bias was induced by the match between histopathology slides and MR images. Likely there was a varying degree of difference in orientation of slicing between post-resection specimens and MRI images. However, this side-by-side comparison could be further improved by using agar fixation of the resection specimen in order to obtain thinner slices as demonstrated in Fig. 1. An important disadvantage of using agar fixation is the lack of availability of obtaining tissues for biological studies. Third, histopathological estimation of tumour composition is not perfectly objective. However the overall tumour subtype classification was concordant with the central review. Furthermore the interobserver variability of our method was tested for the whole-tumour approach. According to previous studies, a single-slice approach is subject to slightly broader reader variability [11]. Finally, we used the enhancement of the erector spinae muscles as a threshold filter for excluding less-enhancing portions of lesions. Formal perfusion analysis could further improve the selection of viable areas of the lesions.

Prospective studies with a larger cohort of patients could further elucidate the potential additional role of DWI and ADC measurements in pretreated nephroblastoma.

## Conclusion

Side-by-side comparison of ADC measurements and histopathology was feasible in the majority of our patients with nephroblastoma. Our pilot study showed a strong linear relationship between the percentages of stromal and blastemal histopathology with ADC parameters.
